# Investigation on physical and physiological properties of extracellular vesicles derived from *Enterococcus faecalis*

**DOI:** 10.1007/s00253-025-13690-0

**Published:** 2026-01-12

**Authors:** Jung-Ah Cho, Sang-Soo Jeon, Go Woon Choi, Chang-Hun Lee, Sung-Jae Kim

**Affiliations:** 1 Department of Orthopedic Surgery, College of Medicine, Hanyang University, Seoul, Republic of Korea; 2 College of Transdisciplinary Studies, School of Undergraduate Studies, Daegu Gyeongbuk Institute of Science and Technology, Daegu, Republic of Korea; 3 Department of New Biology, Daegu Gyeongbuk Institute of Science and Technology, Daegu, Republic of Korea; 4 Foot and Ankle Division, Department of Orthopedic Surgery, Hanyang University Guri Hospital, Guri, Republic of Korea

**Keywords:** Bacteria membrane vesicles, Enterococcus faecalis, Cytokine profiles

## Abstract

Extracellular membrane vesicles (EVs) are nanosized particles that contain various molecules originating from their parental cells and are produced by all three domains of life, including bacteria. Bacterial EVs are known to contribute to bacterial infections and immune responses in various human diseases. *Enterococcus faecalis* is an opportunistic pathogen. In this study, we examined the physical and physiological properties of EVs generated by *E. faecalis*, including particle size, protein composition, and cytokine-inducing profiles. To this end, we isolated EVs from bacteria under different preparation processes, and also a certain condition with the addition of EGCG. First, the bacterial culture supernatants were directly ultracentrifuged (named “Rough”), or filtered through 0.45- or 0.22 µm pore-sized membrane filters (named as “0.45 µm” or “0.22 µm,” respectively). EVs from EGCG-treated bacteria were prepared using a 0.45 µm pore-sized membrane filter and named “EGCG + 0.45 µm.” Each EV sample was subjected to DLS, SDS-PAGE, and cytokine array analyses. DLS results showed that the differently prepared EVs had distinct size distributions depending on the filtration process. SDS-PAGE results revealed unique protein profiles that differentiated EVs under each condition. Treatment of macrophages with each EV sample markedly increased cell viability and size. The cytokine profiles produced by macrophages in response to each EV preparation revealed both common and distinguishable factors. This study has significance in revealing aspects of the biological characteristics of EVs produced by *E. faecalis*, which have previously been largely unknown.

## Introduction

Living organisms across all three domains secrete extracellular membrane vesicles (EVs), which are nanosized particles containing various molecules derived from their parental cells, including proteins, lipids, and nucleic acids. Compared to EVs derived from eukaryotic cells, bacteria-derived EVs (BEVs) have only recently been recognized. Both Gram-positive and Gram-negative bacteria release BEVs, but the mechanisms of BEV biogenesis differ between them. In Gram-negative bacteria, these vesicles are referred to as outer membrane vesicles (OMVs), while in Gram-positive bacteria, they are called cytoplasmic membrane vesicles (CMVs)(Villageliu et al. 2022). Despite their distinct mechanisms of formation, BEVs generally carry physiologically important components characteristic of their bacterial origin. In many bacterial species, BEVs have been implicated in host–pathogen interactions, biofilm formation, horizontal gene transfer, and the secretion of virulence factors. Therefore, BEVs function as important mediators of communication both with host cells and among bacterial communities.

Emerging evidence suggests that BEVs are involved in various human diseases, including autoimmune disorders, allergies, and inflammatory conditions by promoting bacterial infections and modulating immune responses (Wang et al. 2023). Pathogens can evade host immune responses and achieve efficient infections through the actions of BEVs. At the same time, BEVs from pathogenic bacteria contain pathogen-associated molecular patterns (PAMPs) that can activate innate immune cells, as well as antigens capable of inducing B and T cell responses (Peregrino et al. 2024). These properties indicate the potential of BEVs as vaccine platforms.

*Enterococcus faecalis* is a Gram-positive bacterium that resides as a commensal bacterium in the human gastrointestinal tract and is also found in healthy individuals (de Almeida et al. 2018). However, *E. faecalis* is also an opportunistic pathogen capable of causing a variety of human diseases such as endocarditis, sepsis, urinary tract infections (UTIs), meningitis, and other infections (Murray 1990; Hidron et al. 2008). In addition, *E. faecalis* is the most prevalent enterococcal species associated with biofilm-related infections, which are difficult to eradicate with antibiotic treatment (Woitschach et al. 2022). In 2021, it was first reported that *E. faecalis* releases EVs (Afonina et al. 2021). Since then, little has been known about EVs from *E. faecalis*, except that they promote apical periodontitis and exert pro-inflammatory effects (Ma et al. 2024; Niu et al. 2024).

We previously reported the biological and molecular characteristics associated with the biofilm of an isolate of *E. faecalis*, and demonstrated that the phytochemical EGCG affected the biofilm-forming ability and protein composition of the microbe (Cho et al. 2024a, b; Cho et al. 2022). In this study, we examined EVs originated from *E. faecalis*. EVs were isolated from the bacteria cell culture supernatants using three different preparation processes: directly ultracentrifuged, or membrane-filtered with two different pore sizes (0.22 µm or 0.45 µm) prior to ultracentrifugation. Additionally, EVs from *E. faecalis* cultured in the presence of EGCG were prepared by ultracentrifugation after filtration through a 0.45 µm pore-sized membrane. The isolated EVs were then subjected to investigate the particle sizes, protein compositions, and cytokine-inducing properties.

## Materials and methods

### Bacteria culture and EV extraction

*Enterococcus faecalis* was obtained from the National Culture Collection for Pathogens (NCCP, Korea) (https://nccp.kdca.go.kr/main.do), which was established as a national pathogen resources bank in order to promote R&D in the prevention, diagnosis, and treatment of infectious human diseases. *Enterococcus faecalis* (NCCP 15611) is described as being isolated from blood in the Jeollabuk-do region of South Korea, belonging to sequence type (ST) 179 and characterized by resistance to clindamycin, erythromycin, tetracycline, and trimethoprim-sulfamethoxazole. Bacteria were cultured on tryptic soy agar (TSA, Difco) or tryptic soy broth (TSB, Difco). A primary bacterial culture was prepared by inoculating a single colony from on TSA plates into TSB media in a conical tube (SPL Life Sciences, Korea), followed by incubation overnight at 37 °C with shaking at 120 rpm. For EV extraction, the primary culture was diluted with fresh broth to achieve an optical density (OD) at 600 nm (OD600) of 0.1, as measured using a spectrophotometer, and then cultured overnight at 37 °C with shaking at 120 rpm. The overnight culture was subjected to centrifugation at 5000 g for 30 min to separate the cell pellet and culture supernatant. The resulting supernatant was subjected to three different preparation procedures; (1) unfiltered (designated “Rough”) or (2) filtered with a 0.22 µm or 0.45 µm syringe membrane filter (Merck Millipore, Darmstadt, Germany), named “0.22 µm” or “0.45 µm,” respectively. Both unfiltered and membrane-filtered supernatants were concentrated using a 100-kDa cutoff filter unit (Amicon® Ultra-15, UFC910024, Merck Millipore) and then finally ultracentrifuged at 100,000 g for 1 h to obtain the EV pellet. The pellets were washed with DPBS by ultracentrifugation at 100,000 g for 1 h and resuspended in 100 µl of DPBS. For EGCG treatment, EGCG was added at a final concentration of 1 mM during subculturing, and the resulting overnight culture supernatant was filtered through a 0.45 µm syringe membrane prior to ultracentrifugation. The quantity of isolated EVs was determined using a BCA protein assay (Pierce™ BCA Protocol Assay kit, Thermo Scientific™ 23227).

### Dynamic light scattering (DLS) analysis

The measurement of EV sizes using DLS was performed as previously described (Lyu et al. 2021). The extracted EVs were diluted to a concentration of 100 µg/ml in DPBS and then 100 µl of the diluted EV solution was transferred into a cuvette. After stabilizing the laser and temperature equilibrium of the device (Dynapro Nanostar, WYATT Technology), the EV-containing cuvette (NanoStar MicroCuvette Kit) was placed into the DLS device for measurement. For reproducibility and standardization, the following parameter settings were used: laser wavelength (nm), 663.87; temperature controlled, yes; peak radius low cutoff (nm), 0.5; peak radius high cutoff (nm), 5000; auto-attenuation time limit (s), 60; calculate D10/D50/D90, yes; calculate polydispersity, yes; set temperature (C), 25; and wait (min), 5. The size or homogeneity of the EVs was recognized through the radius or the % Intensity, respectively. The measurements were conducted with 10 acquisitions of 5 s each.

#### SDS-PAGE

Equal amounts of each EV preparation were loaded onto a 15% acrylamide-containing gel and electrophoresed at 80–150 V for 2 h. After SDS-PAGE, the gel was stained with Coomassie brilliant blue staining solution (Bio-Rad), followed by destaining with methanol/acetic acid-containing solution. Finally, the gel was placed on a light plate to acquire the gel image.

### Macrophage morphology analysis and cytokine array

A murine macrophage cell line RAW264.7 was purchased from Korean Cell Line Bank (KCLB) and cultured in DMEM supplemented with 10% fetal bovine serum (FBS). To deplete bovine-derived EVs, the FBS was ultracentrifuged overnight at 100,000 × *g* prior to use. The cells were seeded on a 24-well plate at a density of 2 × 10^4^ cells/well and fed with 5 µg of each EVs or 1 × 10^8^ CFU of bacteria cells 24 h post seeding. After a 24-h incubation, the cell culture supernatants were collected for cytokine array analysis, and the remaining cells were observed under a light microscope and assessed for cell viability.

Cell images were captured from 3 different fields at ×40 magnification and analyzed for morphological parameters, including cell size, using ImageJ software. For the cell viability assay, cells were reacted with CCK-8 solution (Dojindo) according to the manufacturer’s protocol, and the measures of percentages were calculated by dividing the absorbance of each experimental group by that of the untreated control group, and multiplying by 100.

Collected cell supernatants were centrifuged to remove cell debris and then applied to a cytokine array (AAM-CYT-3, RayBiotech), followed by the manufacturer’s protocol. Finally, the membrane images were obtained by Luminorph software and analyzed with ImageJ software. Statistical significance was determined by *t*-test. The Venn diagrams were constructed utilizing the following web-based tool: https://www.bioinformatics.com.cn/srplot.

### Bacterial adhesion to hydrocarbon (BATH) test

Bacteria cells were inoculated in 4 mL of TSB and incubated for 24 h at 37 °C. After washing with PBS, the resuspended overnight bacterial culture was measured for the absorbance at 600 nm [A] in triplicate, and then added with decane (SIGMA-ALDRICH D901), followed by thoroughly vortexing for 1 min. After standing for 15 min at RT, the upper decane-containing phase was removed, and the absorbance of the remaining clear phase was measured at 600 nm [B] in triplicate. Results were analyzed using the following equation:

$$Hydrophobicity\left(\%\right)=\frac{A-B}{A}\times 100$$

Hydrophobicity below 20%, between 20 and 50%, and above 50% was defined as hydrophilic, moderately hydrophobic, and highly hydrophobic, respectively.

### Detection of bacterial cell membrane depolarization

The bacterial cell membrane potential was determined as previously reported (Swain et al. 2019). Briefly, the bacterial cell pellet from the overnight culture was resuspended in HEPES-containing buffer and incubated with DiSC3(5) probe (Invitrogen) in the dark at 37 °C, followed by EGCG addition. The fluorescence intensity was measured at 2 min intervals using SpectaMAX (Tecan) at excitation/emission wavelengths of 630/680 nm, respectively.

### Evaluation on biofilm formation

One or 5 µg of untreated or EGCG-treated EVs were added to the 5 mixed bacteria culture in a 14-ml round-bottom tube. After incubation at 37 °C for 24 h, the bacterial culture media was removed and washed with PBS 3 times. The biofilm formed on the tube surfaces was quantified with crystal violet assay as previously described (Cho et al. 2022).

## Results

### Size distribution and protein compositions of EVs from *E. faecalis*

Compared to the standardized EV isolation methods used for animal cells, the procedures for isolating BEVs tend to vary considerably across studies (Lee et al. 2007, 2009; Codemo et al. 2018; Thay et al. 2013). One major difference lies in the filtration step, where either 0.22 µm or 0.45 µm filters were employed. Given the heterogenous nature of EVs, we first aimed to compare bacterial EVs isolated by different methods. To this end, the bacterial culture supernatants were divided into three batches; one was left unfiltered, while the others were filtered through 0.22 µm or 0.45 µm syringe membranes, respectively. After concentration with Amicon filtration, EV pellets were obtained from each supernatant by ultracentrifugation and finally resuspended in PBS, named as “rough” for the unfiltered sample, and “0.22 µm” or “0.45 µm” for the filtered samples depending on the membrane pore size used.

**Fig. 1:**
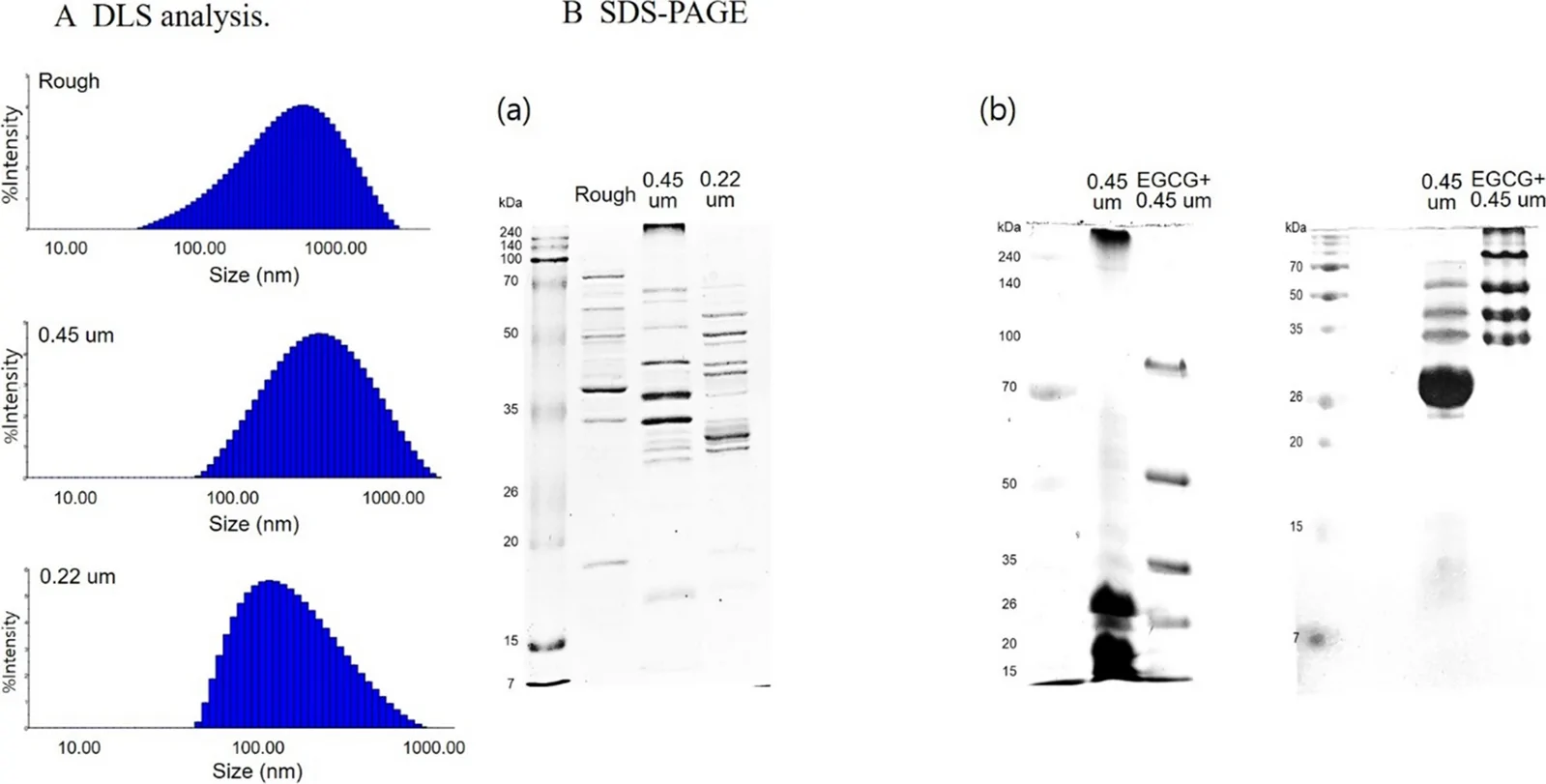
Physical and molecular analysis on EVs. **A** DLS analysis of EVs isolated without filtration (Rough) or following filtration through 0.45 µm or 0.22 µm pore size membrane. **B** SDS-PAGE of EVs. (a) Comparison of protein profiles of EVs that were extracted directly (Rough) or after filtration with 0.45 µm or 0.22 µm pore size membrane. (b) Comparison of EVs filtered through 0.45 µm membrane with or without EGCG treatment

With the EVs, we first analyzed heterogeneity in particle size using dynamic light scattering (DLS) (Fig. 1A). The Rough EVs exhibited a wide size distribution, ranging from 97.305 to 866.945 nm, with a mean diameter of 612.62 nm. On the other hand, 0.45 µm EVs showed a narrower distribution (93.29 to 520.17 nm, mean 426.39 nm). In the case of 0.22 µm EVs, the particle size heterogeneity was much less than that of Rough or 0.45 µm, as observed at sizes from 34.522 to 240.077, with a mean of 177.49 nm. Then, we analyzed the protein composition of each EV preparation via SDS-PAGE. As shown in Fig. 1B, the three differently prepared EVs exhibited a unique protein band pattern that was distinct from each other. These data indicated that different EV preparation methods may result in different physiological activities.

### Immune cell stimulation by EVs from *E. faecalis*

**Fig. 2:**
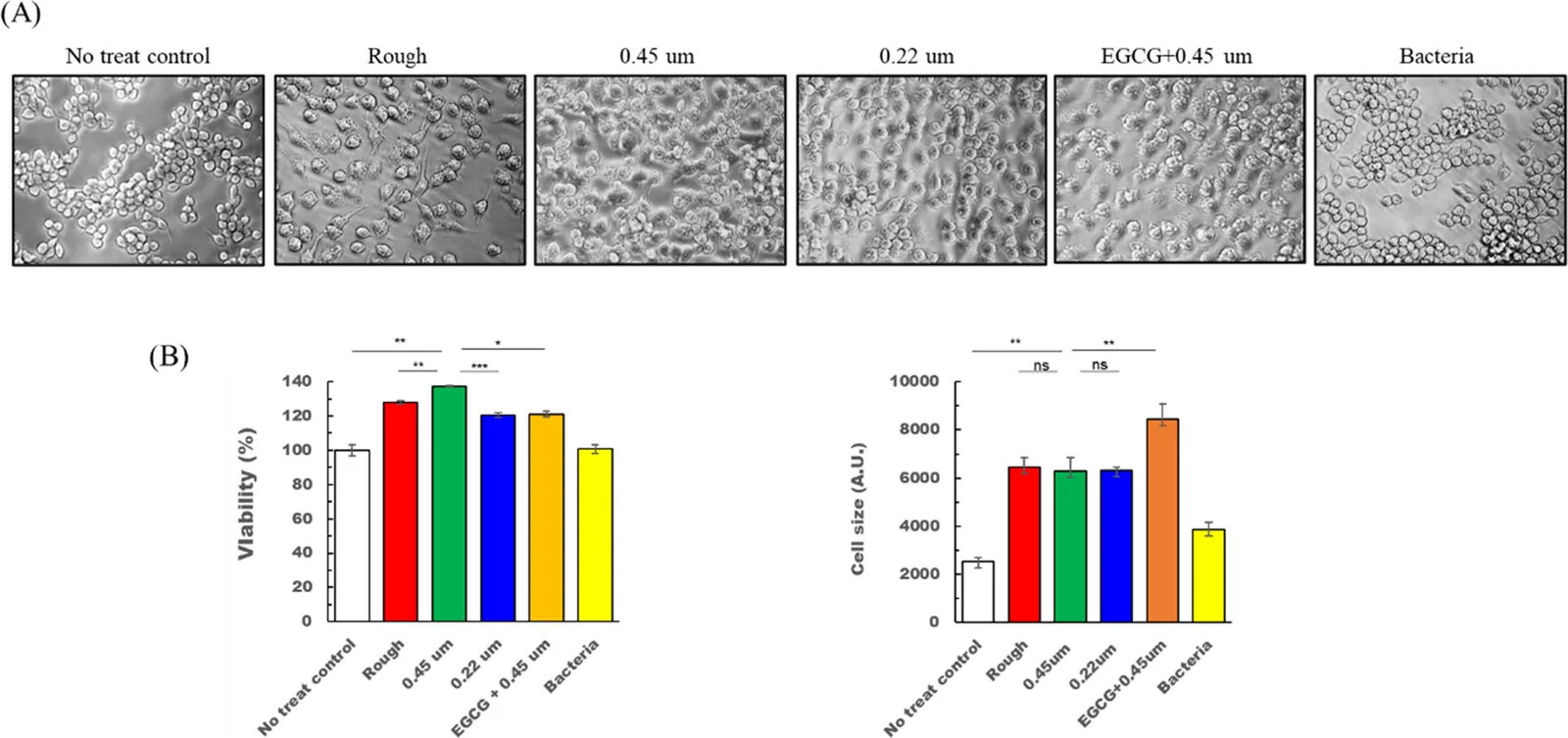
The biological effects of EVs on macrophage cells. **A** Representative cell images. **B** Cell viability (left) and cell size (right). Asterisks indicate statistical significance; ns for no significance, * for *P* < 0.5, ** for *P* < 0.05, *** for *P* < 0.001

**Fig. 3:**
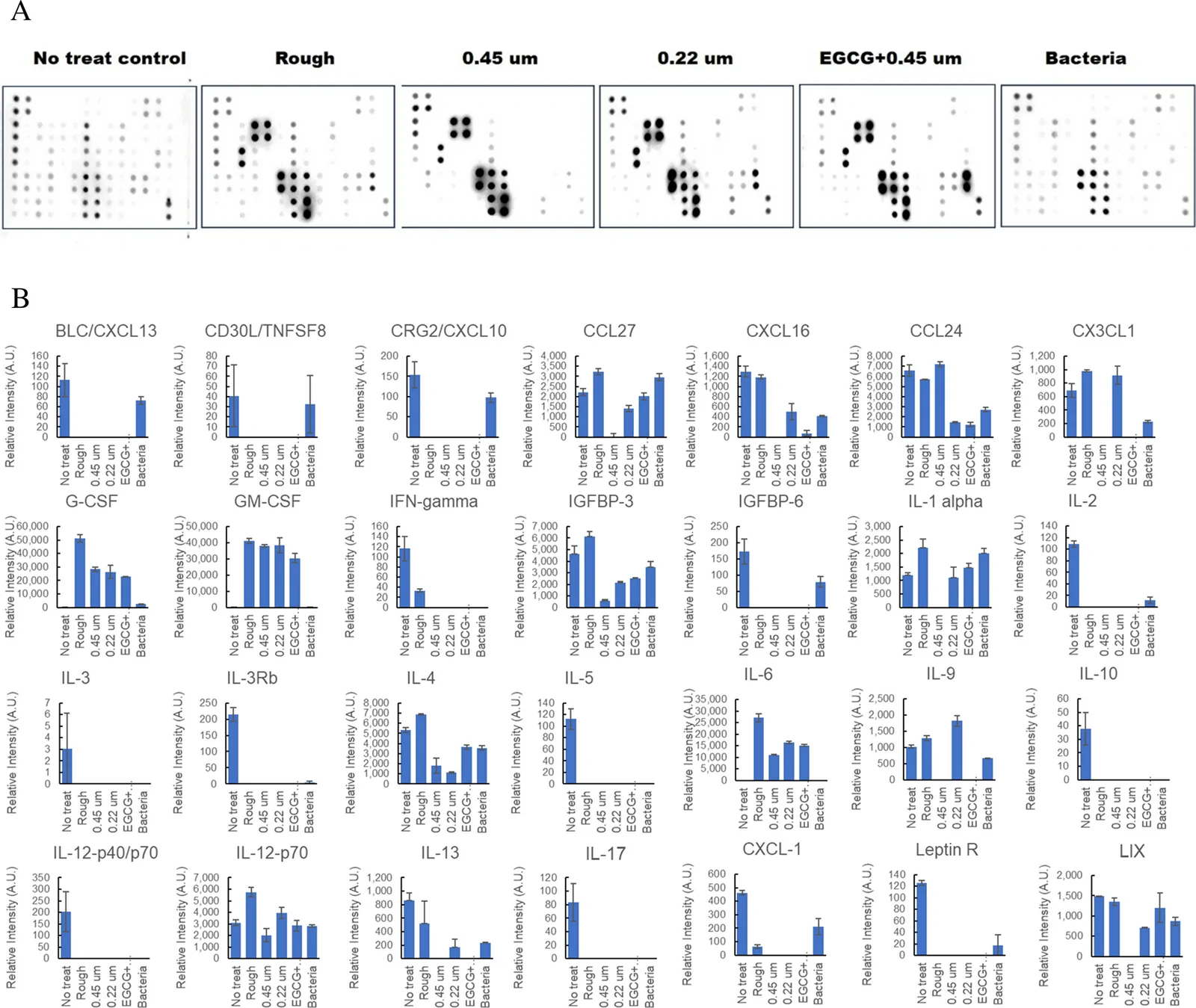

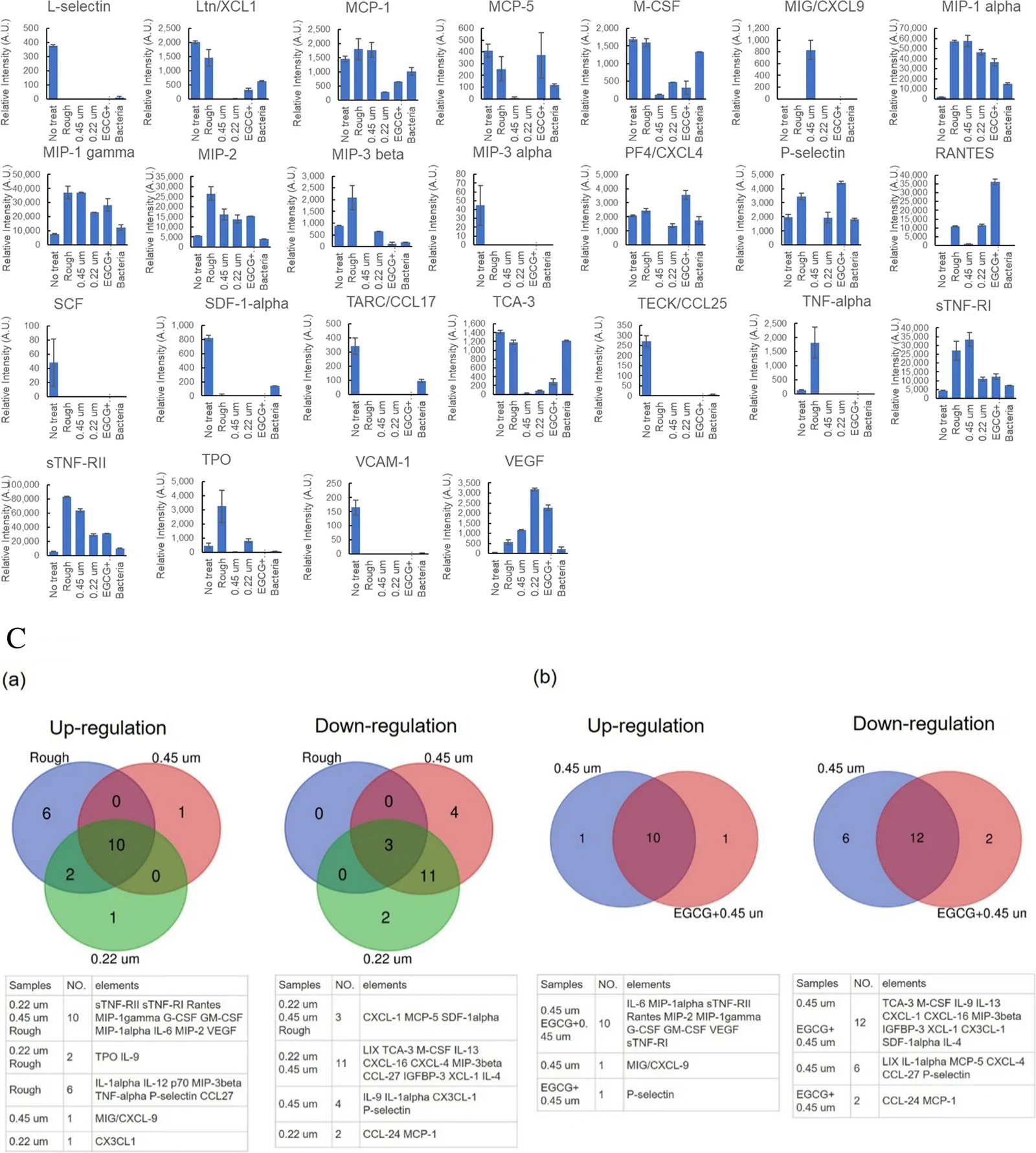
Cytokine array. **A** Representative blot images. **B** Quantification graphs. **C** Venn diagrams. (a) Rough vs. 0.45 µm vs. 0.22 µm, (b) 0.45 µm-filtered untreated vs. EGCG treated EVs

As a representative physiological activity of EVs, we aimed to test the immunoregulatory properties of each EV. For that purpose, macrophages were utilized because they are the first-line defenders against bacterial attack. We added each EV to cultured macrophage cells and incubated them for 24 h. Subsequently, we observed cell morphological changes and assayed the cells for viability (Fig. 2) after collecting the culture supernatant for cytokine profiling (Fig. 3). When macrophage cells were fed with each EV, it was revealed that all the EVs from *E. faecalis* significantly enhanced the cell viability compared to the no treat control group, with 0.45 µm EVs showing the most potent effect. In addition, the cell sizes tended to be larger in all the experimental groups with EVs than in the no treat control group and even in the bacteria cell treat group. There was no statistical significance between EV-treated groups. The findings for the increase of cell viability and size may indicate in this context that *E. faecalis*-derived EVs induced macrophage activation and survival with no cytotoxicity.

The cell supernatant from the macrophage cells that were reacted with each EV was subjected to cytokine array for cytokine production profiling (Fig. 3). The qualitative blot images (Fig. 3A) were quantified using ImageJ software (Fig. 3B), and the cytokines with intensity values below 200 were excluded from further analysis (Fig. 3C). As shown in Fig. 3C, EV treatments modulated cytokine expression in both common and distinct patterns, depending on the cytokine. For example, Rantes, MIP-1 gamma, G-CSFs, etc. were upregulated by all the EVs, whereas IFN-gamma, CXCL-1, etc. were consistently downregulated across all EV treatments. In contrast, IL-9 was upregulated by Rough and 0.22 µm-filtered EVs, but downregulated by 0.45 µm-filtered EVs. MIP-3beta and CCL-27 showed the most prominent filtration-dependent effects, being downregulated by filtered EVs but upregulated by unfiltered (Rough) EVs. Among the filtrated EVs, CX3CL-1 exhibited the most notable differential regulation, being upregulated by 0.22 µm-filtered EVs but downregulated by 0.45 µm-filtered EVs.

### The impact of an external stimulation on EVs of *E. faecalis*

**Fig. 4:**
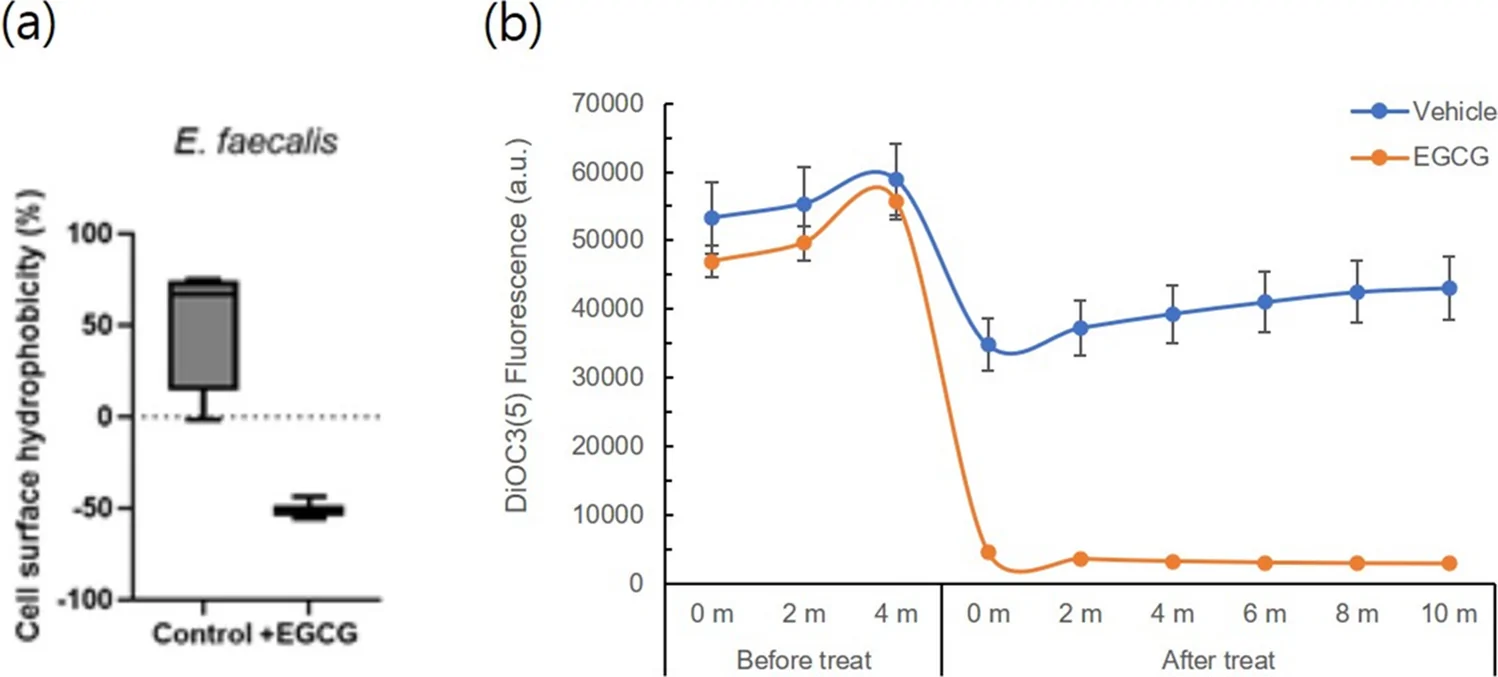
Investigation of the effects of EGCG on bacterial cell membrane integrity. Cell surface hydrophobicity (**a**) and membrane potential (**b**)

In our previous studies, we demonstrated that the phytochemical EGCG strengthened the biofilm-forming ability of *E. faecalis* and altered the protein composition within the biofilm (Cho et al. 2022; Cho et al. 2024a, b). In the present study, we explored the effects of EGCG on EV-related characteristics of *E. faecalis*. We first examined the possibility that EGCG could modulate EV yield. Given that EVs are released through the bacterial cell membrane, we hypothesized that changes in cell membrane integrity would occur if EV production levels were regulated by EGCG. To test the hypothesis, we examined two representative membrane-associated properties: cell surface hydrophobicity and membrane potential (Fig. 4). Assessment of cell surface hydrophobicity was performed using the bacterial adhesion to hydrocarbons (BATH) test with decane. The results revealed that EGCG dramatically modulated the surface property of cells, shifting from mild hydrophobic to more hydrophilic (Fig. 4a). To measure membrane potential, bacterial cells were labelled with a membrane potential-sensitive DiSC3(5) dye after EGCG treatment. The result showed that EGCG significantly reduced the bacterial cell membrane potential (Fig. 4b).

Next, we compared the protein composition of EGCG-treated EVs with that of untreated EVs. To isolate EGCG-treated EVs, the bacterial culture supernatant was filtered with a 0.45 µm membrane before ultracentrifugation because the result with DLS analysis in Fig. 1A indicated that 0.45 µm membrane-filtered EVs were the most homogenous within a relatively less size spectrum. As shown in Fig. 1B (b), EVs treated with EGCG displayed a markedly distinct protein profile compared to untreated EVs. To evaluate the functional effects of EGCG-treated EVs on macrophages, we examined cell viability and morphology following a 24-h treatment. While EGCG-treated EVs induced a comparable level of cell viability as untreated EVs, they led to a significant increase in cell size (Fig. 2). In terms of cytokine production, EGCG-treated EVs caused distinct changes in the production of some cytokines (Fig. 3). In particular, Rantes was the most prominently increased (Fig. 3A and B), and P-selectin was solely induced by EGCG treatment (Fig. 3C (b)).

**Fig. 5:**
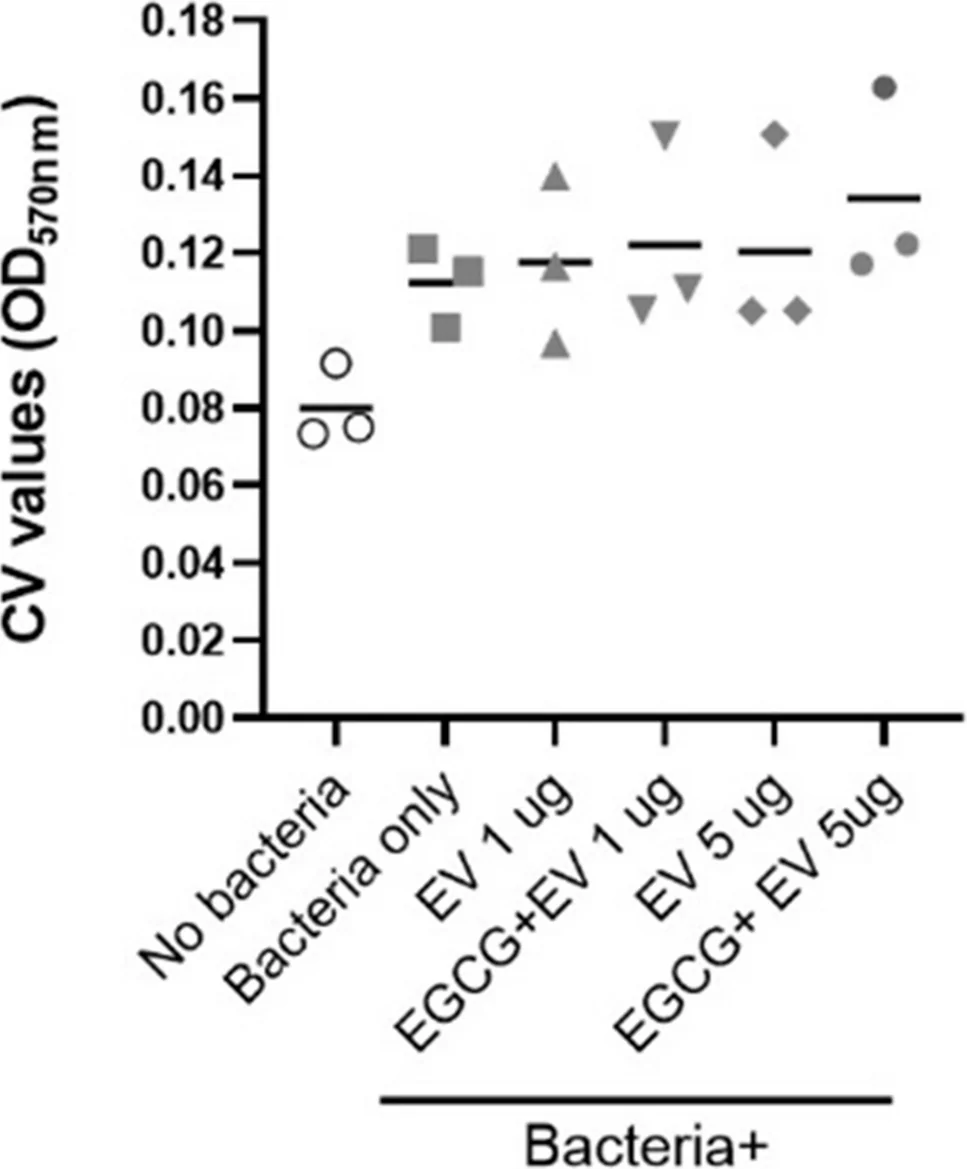
Quantification of biofilm formed by 5 mixed bacteria in the presence of untreated or EGCG-treated EVs (1 or 5 µg) of *E. faecalis*

We finally tested whether EVs from *E. faecalis* could influence life forms of other cells, especially focusing on biofilm formation. For this purpose, we added the untreated or EGCG-treated EVs of *E. faecalis* to five different mixed cell cultures consisting of *Staphylococcus aureus*, *Pseudomonas aeruginosa*, *Staphylococcus epidermidis*, *Streptococcus mitis*, and *Klebsiella pneumoniae*. The five bacteria used in this experiment were randomly selected based on our previous study (Cho et al. 2022). The result showed that 5 µg of EGCG-treated EVs resulted in a mild increase in the level of biofilm formation of the bacteria mixture (Fig. 5).

## Discussions

Extracellular vesicles are important cell messengers that are utilized for cell communication throughout all living organisms, including bacteria (Gill et al. 2019). Compared to evidence that has been intensively accumulated for EVs from animal cells, our understanding of EVs from bacteria is relatively limited. Notably, Gram-negative and Gram-positive bacteria employ distinct mechanisms for EV biogenesis (Ho et al. 2024), and even a single bacterium may have unique molecular contents in their EVs. Therefore, there are still a lot to be revealed.

During the intensive searches for bacterial EVs, we found that the isolation protocols have not been well standardized. In particular, when filtering bacterial culture supernatants for EV preparation, some studies used 0.22 µm filter membranes, while others used 0.45 µm membranes, even for the same bacterial species (Im et al. 2017; Lee et al. 2009; Wang et al. 2018). We believe that differently prepared EVs would exert different biological properties, as EVs are very heterogeneous particles. Thus, we attempted to explore the physical and physiological differences of the EVs according to their preparation methods.

Regarding EVs from *E. faecalis*, there were two researches recently published in 2024 (Niu et al. 2024; Ma et al. 2024). The study by Zhao et al. used 0.45 µm first and then 0.22 µm membrane filters for EV preparation (Ma et al. 2024), where the mean diameter of EVs was approximately 165 nm. The other study by Ran S. et al. prepared EVs from *E. faecalis* using a 0.22 µm pore size filter, where the mean diameter of the EVs was about 135 nm or 199 nm at pH 9 or 7, respectively (Niu et al. 2024). In our study, it was revealed that the sizes of EVs filtered with a 0.22 µm pore size membrane were composed of particles with a mean of 177 nm, which falls within the range reported by the previous reports. Since EVs are highly heterogenous, their characteristics may vary from bacteria to bacteria (Sarra et al. 2020; Dell'Annunziata et al. 2020). Therefore, the analysis of EV size of bacteria under diverse conditions could be a research topic in itself.

Immunological aspects of EVs derived from *E. faecalis* have been partially elucidated also in the previous studies by others as mentioned above (Niu et al. 2024; Ma et al. 2024). In their studies, EVs derived from *E. faecalis* promoted M1 polarization of macrophages and induced the release of pro-inflammatory cytokines such as IL-1β, IL-6, and TNF-α. In our present study, we observed that IL-6 levels were consistently elevated by all kinds of EVs tested, while IL-1β was undetectable, and TNF-α was increased only by Rough EVs. These findings suggest that IL-6 may serve as the most consistent and representative indicator for evaluating the inflammatory potential of *E. faecalis* EVs.

In addition to IL-6, this study revealed that EVs from *E. faecalis* increased the secretion of MIP-1a, sTNF-RI, sTNF-RII, Rantes, MIP-2, MIP-1γ, G-CSF, GM-CSF, and VEGF. The finding for the increase of VEGF secretion was interesting because it was known that VEGF production from macrophages was enhanced by anti-inflammatory stimuli (Wu et al. 2009) and promoted M2 polarization of macrophages (Wheeler et al. 2018). Therefore, it was suggested that EVs from *E. faecalis* may possess not only pro-inflammatory but also anti-inflammatory effects, which are modulated depending on surrounding environments.

On the other hand, the levels of CXCL-1 and SDF-1alpha (also known as CXCL12), both recognized as angiogenic chemokines (Darakhshan et al. 2019), may indicate a potentially suppressive role of EVs from *E. faecalis* on vasculogenesis during the immune responses. It was also interesting that the pattern between M-CSF and G-/GM-CSF production was contrasting. The result suggests that *E. faecalis* EVs may influence the differentiation and function of myeloid cells, possibly exerting paracrine effects on granulocytes via macrophage modulation.

Epigallocatechin gallate (EGCG), a major constituent of green tea extract, is known for its anti-bacterial effects (Ikigai et al. 1993; Steinmann et al. 2013). However, we previously discovered that EGCG enhanced the biofilm-forming ability of *E. faecalis*, increasing the inclusion of virulence-related proteins into the biofilm (Cho et al. 2024a, b). The findings inspired us to hypothesize that EGCG might also affect EV biogenesis, as EVs are known to carry virulence factors of pathogens. As expected, we found in this study that EGCG could alter the characteristics of bacterial cell membrane and wall, including cell surface hydrophobicity and membrane potential, which are potential factors involved in EV biogenesis. The results of the altered cell surface hydrophobicity and cell membrane potential by EGCG indicated that EV production could be accelerated or inhibited by altered membrane dynamics by EGCG. In addition, EGCG contributed to changes in the molecular composition and physiological properties of EVs, as was also observed in our previous study (Cho et al. 2024a, b) with the bacteria themselves.

The changes in cytokine profiles by EGCG-treated EVs from *E. faecalis* were interesting. P-selectin was the most discriminatory factor in distinguishing EVs with from EVs without EGCG, as it was downregulated by EVs without EGCG, but markedly upregulated by EVs with EGCG. For CXCL-9, it was prominently upregulated by EVs without EGCG, whereas EVs with EGCG failed to induce its production. Rantes was also a notable molecule. Although both EVs without and with EGCG induced its production, EVs with EGCG significantly increased its production. In contrast, CCL-24 and MCP-1 production were suppressed by EVs with EGCG, while EVs without EGCG exerted no effect on them.

To our knowledge, this is the first study to elucidate the cytokine profiles induced by different fractions of EVs generated by *E. faecalis*. Nevertheless, there are still many more questions that need to be revealed through further studies. We will continue our investigation into more details on the mechanisms of biological and immunological activities to accumulate helpful information for overcoming the pathogenic *E. faecalis*.

Taken together, this study demonstrated that *E. faecalis*-derived EVs differ in their physical and functional characteristics depending on the preparation method and culture conditions. Each EV preparation exhibited distinct physicochemical and immunological features, reflecting differences in vesicle size, composition, and macrophage responses. Rough EVs, while heterogeneous, may capture the full spectrum of bacterial secretions and are suitable for exploratory analyses. In contrast, 0.45 µm- and 0.22 µm-filtered EVs represent more defined populations, with the latter enriched for small vesicles ideal for molecular characterization. EGCG-treated EVs showed distinct protein and immune profiles, suggesting that external stimuli can modulate EV cargo and host interactions. These findings underscore the functional diversity of bacterial EVs and highlight the importance of method selection for their research and therapeutic applications.

## Data Availability

No datasets were generated or analysed during the current study.
